# Functional Capabilities of the Earliest Peptides and the Emergence of Life

**DOI:** 10.3390/genes2040671

**Published:** 2011-09-26

**Authors:** E. James Milner-White, Michael J. Russell

**Affiliations:** 1 College of Medical, Veterinary and Life Sciences, Glasgow University, Glasgow G128QQ, UK; 2 Jet Propulsion Laboratory, California Institute of Technology, Pasadena, CA 91109, USA; E-Mail: mrussell@jpl.nasa.gov

**Keywords:** catgrips, emergence of life, hydrothermal mound, nests, niches, peptides

## Abstract

Considering how biological macromolecules first evolved, probably within a marine environment, it seems likely the very earliest peptides were not encoded by nucleic acids, or at least not via the genetic code as we know it. An objective of the present work is to demonstrate that sequence-independent peptides, or peptides with variable and unreliable lengths and sequences, have the potential to perform a variety of chemically useful functions such as anion and cation binding and membrane and channel formation as well as simple types of catalysis. These functions tend to be performed with the assistance of the main chain CONH atoms rather than the more variable or limited side chain atoms of the peptides presumed to exist then.

## 1. Introduction

**Figure 1:**
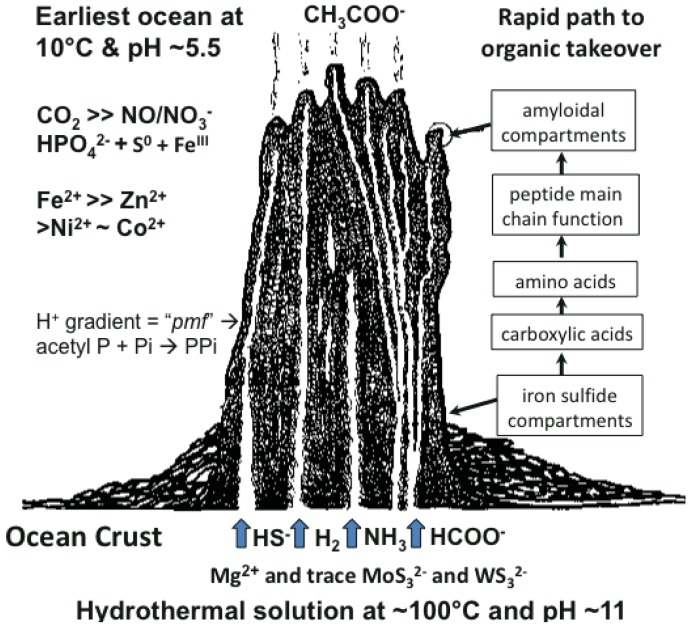
Cartoon of model low entropy environment for the emergence of metabolism via hydrothermal hydrogenation of oceanic CO_2_, amination of carboxylic acids and the condensation to disordered peptides. These reactions are hypothesized to take place in the outer porous iron sulfide-bearing walls to a submarine hydrothermal mound (see boxes on right of figure). Thermodynamic energy is boosted by the ambient proton-motive force. This force is assumed to have driven pyrophosphate generation, augmenting pyrophosphate of the substrate type formed by catalysis [7,8,20]. Amyloidal peptides eventually take over the role of compartment walls while retaining and nesting inorganic clusters of metals, metal sulfides and phosphate and allowing the transfer of potassium and water across these first membranes/cell walls [16,17]. In this way the properties both of the semipermeable and semiconducting membranous walls and of the catalytic propensities of the inorganic entities improve [21,22]. In this model the emergence of protometabolism from aqueous geochemistry predates transition to the RNA world [10,14,35].

Many ideas about the emergence of life have been presented [1-6]. The scenario we favor takes cognizance of the need of materials and a continual supply of energy at the appropriate magnitude to build a hatchery for life to onset. This hatchery would sustain the first metabolizing compartments and, eventually, a burgeoning population of cells [7-10]. A requirement of emergent structures is a low entropy condition whereby order in one system begets order in the next [11]. In this case, the order bestowed on emergent life and its factory is contributed by a chemostated (pH = 10 ± 1 unit) and thermostated (T = 70 °C ± 30 °C) submarine hydrothermal spring operating for tens of thousands of years [12]. The hydrothermal solution bears hydrogen as fuel, ammonia for aminations, sulfide for compartment structure and molybdenum and tungsten for catalysis [13]. Interaction of these spring waters with the early protonic and carbonic ocean, with its load of transition metals and minor concentrations of phosphate, not only produces an edifice of porous mineral precipitate but also induces a proton gradient across the margins that acts as a natural proton-motive force to drive a variety of condensations (Figure 1) [7,8]. Pore spaces on the margins of the growing hydrothermal edifice act as low entropy compartments where organic molecules are synthesized through the hydrogenation of the CO2 invading the mound margins, catalyzed by transition metals within the compartment walls (Figure 1). Products are aminated and polymerized by pyrophosphates condensed from monophosphate, perhaps with acetyl phosphate, by the ambient proton-motive force [14,15]. The inorganic compartments comprise a low entropy hatchery of life where organic reactants are forced to interact through their very proximity at low water activity [16,17]. Amino acids generated in this milieu may be condensed into peptides [18,19].

**Figure 2:**
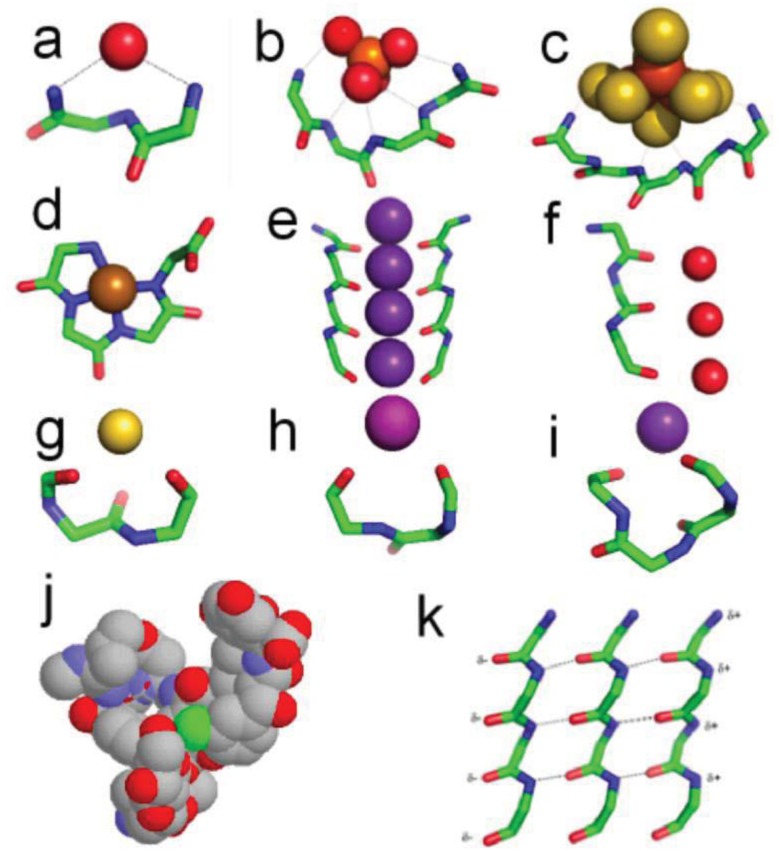
Motif structures (**a**) nest binding a carbonyl oxygen. (**b**) nest binding a phosphate ion. (**c**) nest binding a Fe_4_S_4_ iron-sulfur center. (**d**) covalent Ni^+^-tetrapeptide complex—a peptide pigment. (**e**) two of the four peptides forming the selectivity filter of the potassium channel. (**f**) the peptide forming the aquaporin channel. (**g**) catgrip bound to a calcium ion. (**h**) niche3 bound to a K^+^ ion. (**i**) niche4 bound to a K^+^ ion. (**j**) vancomycin, which can be regarded as a nest designed to bind a carboxylate group in the bacterial cell wall (the carboxylate of acetate is seen in sticks and the antibiotic is in spacefill). (**k**) three strands of α-sheet. (note the similarity in conformation with e and f). α-sheet, unlike β-sheet, has an inherent polarity, indicated by the partial charges δ^+^ and δ^−^. In (**a-i**) and (**k**) the side chains are omitted. Colors: carbon, green; oxygen, red; nitrogen, blue; sulfur, mustard; potassium, purple; iron, rust; nickel, brown; chlorine, green; calcium, yellow; phosphorus, orange.

Some of the properties of these peptides, as will be shown, lend themselves to assisting the development of prebiotic systems in ways that are hard to envisage for polynucleotides. They continue offering low entropy sites though at a much smaller scale and therefore more effectively than do the mineral compartments. We note in passing that lipids too are difficult to synthesize under prebiotic conditions and have even less sequestering power than polynucleotides. Moreover, cogent arguments have been advanced that some protein features are the most ancient conserved macromolecular entities that exist [23-28]. Thus, we suggest that, once the mineral hatchery for life was built, the first major biomolecules produced there were peptides that took over the roles of the minerals as compartment walls, and chelated inorganic clusters as precursors of the metal and metal sulfide proteins as well as of the phosphates (Figure 2a) [29,30]. Moreover, a synergy would have existed between peptides on the one hand and metabolic entities on the other [31-33]. This idea does not preclude the existence of an RNA or protein/RNA world but the premise is that any such era came later and was probably derived from the coenzymes [14,34,35]. It seems improbable the earliest peptides consisted of large domains of tightly folded polypeptide chains as in present day proteins. Instead they would have been small, simple and heterochiral in nature. Without a genetic code as we know it, different polypeptide molecules probably had a variety of compositions and sequences and thus lacked defined large-scale three-dimensional structures. Although theoretically not limited to the 20 amino acids in current proteins, amino acid occurrence was governed by ease of synthesis, with a preponderance of glycines and a few others probably in the order alanine > aspartate > valine [6]. These others were almost certainly heterochiral at least initially [36]. The homochirality of present-day amino acids has a great effect on the structures they adopt [37] and the α-helix, especially, is only favored in homochiral peptides. The consequence is that early peptides were more exposed to solvent water and variable and motile in their 3D structure than present-day evolved proteins. This does not mean they lacked any structure at all, as, especially on the scale of a few Å, recurring features do occur. One aspect is that the variability and unpredictability of the side chains—their stochastic nature—would have caused main chain, rather than side chain, features to be employed for functional purposes. Most of the motifs described below are ones that only employ main chain atoms for various anion or cation binding activities and this aspect would have made them functionally useful during the emergence of metabolism and the onset of life.

A web application, Motivated Proteins [38] gives access to a database of high-resolution proteins in which the motifs described herein, as well as other small ones, can be examined. Motifs can be identified and visualized on their own in atomic detail. Alternatively their situations in relations to the rest of the protein, either in 3D or in relation to the sequence, can be viewed.

## 2. Main Chain Functions of Peptides

### 2.1. Nests Bind Anions

In nests seen in (Figure 2a), three consecutive amino acids typically form a cavity such that the main chain NH groups of the first, second and third residues bridge, via hydrogen bonding, a negatively charged, or partially negatively charged oxygen atom. Commonly the second NH group points away and is not hydrogen-bonded and the pattern of hydrogen bonding varies somewhat, but in general the nest is an anion-binding site [39-44]. The atoms hydrogen bonded to NH groups of the nest are sometimes called eggs. The feature is common such that 8% of residues in all soluble native folded proteins are part of one. Two or more nests can overlap to form a larger and wider cavity that can bind an anionic group instead of just a single atom. The NH groups all point approximately to the center of the curve formed by the polypeptide. One such cavity is observed in the well known ATP or GTP binding site in proteins: the phosphate-binding P-loop, in G-proteins, kinases and ATPases, as in (Figure 2b).

A nest conformation is generated when the ϕ, ψ angles of two successive amino acid residues are approximately enantiomeric, with the defining angles near to those given in Table 1. The ϕ, ψ angles of the third nest residue do not affect the nest conformation. Nests, defined by these angles, are of two kinds called RL and LR. Overlapping nests can be RLR, RLRL, *etc.*, as in (Figure 2b,c). R stands for right-handed (negative ϕ) and L stands for left-handed (positive ϕ). In proteins, 80 percent are RL and 20 percent are LR. In many RL nests the first and third NH groups bridge a proteinaceous oxygen atom whereas most LR nests are not bridged at all and in general are less concave than RL nests.

**Table 1:** Many three-residue motifs are specified by two pairs of ϕ,ψ angles for two successive amino acids. When its main chain atoms are enantiomeric (ϕ_A_=−ϕ_B_ and ψ_A_ = −ψ_B_) a dipeptide is said to be enantiomeric and may occur as part of a longer enantiomeric peptide. The niche4 is a four-residue motif specified by three pairs of angles. In proteins glycines are common (50–80%) at residues where ϕ > 0°.

**Motif**	**ϕ_A_**	**ψ_A_**	**ϕ_B_**	**ψ_B_**	**ϕ_C_**	**ψ_C_**	**Enantiomeric?**
RL nest	−94°	−3°	76°	22°			nearly
LR nest	74°	16°	−77°	−20°			nearly
RL α-strand	−60°	−60°	60°	60°			yes
LR α-strand	60	−60	60	−60			yes
niche3	−91°	−6°	−87°	141°			no
niche4	−73°	−19°	−94°	9°	−99°	134°	no
RL catgrip	−70°	150°	70°	−150°			yes
LR catgrip	70°	−150°	−70°	150°			yes
covalent metal-peptide	180°	0°	180°	0°			yes

Many nests occur as part of other hydrogen-bonded motifs in which the egg atom bound to the nest is a carbonyl oxygen not far away in sequence. However some nests have what are functional rather than structural roles. For example, nests occur within the commonest group of peptide-bond-splitting proteases such as trypsin and chymotrypsin as a feature called the oxyanion hole. This feature bridges, and thereby stabilizes, the negatively charged oxygen atom intermediate that forms from the carbonyl group whose carbon is nucleophilically attacked by a serine side chain of the enzyme.

Vancomycin is a bacterial glycopeptide antibiotic. It acts by binding to a C-terminal D-alanine residue, an intermediate in bacterial cell wall synthesis. In the crystal structure of vancomycin-acetate, the carboxylate of acetate mimics the C-terminal D-alanine carboxylate. (Figure 2j) shows how vancomycin can be regarded as a box with a functional nest at the bottom for binding this carboxylate [38]. The peptidic part of vancomycin consists of alternating L and D amino acids, consistent with heterochiral peptides of this sort that readily form nests. Several other naturally occurring and synthetic peptides are also known to occur as nests [41,45-47].

### 2.2. Phosphates Bind Nests

It would seem that during early evolution nests were used for anion binding and still retain this function in several present-day protein and peptide features. An example is phosphate binding. Whereas the P-loop proteins are thought to be one of the most ancient of protein folds [25-28], phosphate binding in more recently evolved features like inositol triphosphate binding proteins and phosphoserine, phosphothreonine and phosphotryrosine-specific binding proteins and protein kinases employ mainly the positively charged side chains of lysine, arginine or histidine for binding phosphate groups rather than nests [48]. Perhaps the functional nests evolved at a time, before the genetic code, when amino acids with side chains were of sporadic and unreliable occurrence, so features relying on main chain atoms were the more reproducible. The P-loop, the commonest protein feature that binds the β-phosphate of ADP, ATP, GDP or GTP, incorporates an overlapping LRLR pentapeptide nest that may be a relic of the earliest phosphate-binding peptides. FeS proteins where the iron-sulfur center is bound to nests, discussed next, may also be early enzyme relics.

### 2.3. FeS Centers Bind Nests

As discussed above, life is thought to have evolved in submarine hydrothermal mounds growing above alkaline springs exhaling at up to ∼100 °C and rich in H_2_, HS^–^, CH_4_ and NH_3_ (Figure 1). These fluids interface an acidulous carbonic ocean containing Fe^++^, Ni^++^, Zn^++^, Co^++^ and other metal ions [7,8]. Since nucleotides are much more difficult to make than peptides, a period with peptides but no nucleic acids, would have occurred. Another period, with nucleic acid but before the advent of the genetic code, is also likely to have existed [10,14,34,35]. In experiments simulating early evolution the amino acids made are glycine-rich and those with side chains are heterochiral. Peptides with such amino acid compositions tend to form nests.

The sulfide-rich fluid emerging from submarine off-ridge hydrothermal vents at the Hadean ocean floor forms porous precipitates of iron sulfide on meeting the ferrous iron-bearing carbonic Hadean Ocean [16,17,29], and it is here that life may have first emerged and developed [7]. In the light of this it is intriguing that fifty percent of iron-sulfur centers in present-day proteins are bound, via NH^⋯^S hydrogen bonding, to nests, often overlapping ones. If all the sulfur atoms are included, the net charge of any iron sulfur center is negative, whether the iron is Fe^2+^ or Fe^3+^. So they can be regarded as anionic. In some cases there are four overlapping nests as in the example of the iron-sulfur center in (Figure 2c). Iron-sulfur centers are potentially valuable for catalysis via oxidation-reduction reactions since they can exist as Fe^2+^ or Fe^3+^ and the ability to stabilize and sequester these centers as peptide nests must have been, and still is, an advantage. As well as sequestration, the NH groups would tend to stabilize the reduced rather than the oxidized forms. Originally such iron-sulfur centers would have bound alkyl sulfides produced within the hydrothermal mound fluid rather than cysteine side chains [30,49,50].

### 2.4. Potassium Channels

Potassium channels possess a characteristic GYG signature sequence that folds to a similar three-dimensional structure; some of these transporters are about 10,000 times more permeable to potassium and rubidium ions than to sodium ions. Crystal structures [51] of these integral membrane proteins reveal a row of main chain carbonyl groups from adjacent GYG residues arranged in four-fold symmetry around the potassium ions in the narrow part of the channel as in (Figure 2e). The feature is called the selectivity filter. The most decidedly linear part of this row of carbonyl groups is formed by the main chain conformation of the GY residues. The conformation is like that of overlapping nests, but extended, so that the nest concavity is lost. Flattened nest conformations are relatively unusual in native proteins but another channel that includes a functional peptide of this sort is the aquaporin channel [52], illustrated in (Figure 2f). Here the channel has a single peptide and its function is to allow a row of water molecules to be transported across the membrane. These channel conformations are similar to that of the peptides within α-sheet discussed in the next section.

### 2.5. Alpha-Sheet and Amyloid

It is evident that, while most nests in natively folded proteins are concave, some are flattened. As well as occurring in channels, such peptides are similar to those expected in α-sheet, as seen in (Figure 2k), a structure predicted by Pauling and Corey in 1951 [53]. However crystal structures of native proteins revealed little and α-sheet was largely ignored. Although flattened nests are relatively common they are only rarely organized into α-sheet in proteins. Recent work has suggested that α-sheet, shown in (Figure 2k), may be the material of the toxic amyloid precursor and that it converts itself into the somewhat more stable and non-toxic β-sheet of mature amyloid by the process of peptide plane flipping [54-59]. If the amyloid precursor is indeed the material of amyloid that is extremely toxic to cells, the evolution of cells would have selected against its occurrence, explaining why it is relatively rare in native folded proteins.

Amyloid is well known as the proteinaceous substance that is the mediating agent of conditions such as Alzheimer's, Parkinson's, Huntington's, type II diabetes and the prion diseases like CJD (Creuzfeld-Jacob disease). These so called amyloidoses are not caused by microorganisms but rather by harmful misfolded proteins called amyloid. Recently it has been shown that a propensity to form amyloid is not just the property of a few specialized proteins but that ordinary proteins such as myoglobin can also form amyloid in appropriately denaturing environments [60]. This has led to the idea that amyloid may have been the commonest form of early proteins [61]. Mature amyloid is thought to be composed of multiple layers of large β-sheets, probably with parallel strands [62]. However it is the amyloid precursor, rather than the mature form, that is toxic. The nature of this precursor is controversial, but evidence is accumulating suggesting it is α-sheet. Amyloid (whether made of α- or β-sheet) is a sticky and gelatinous substance. It has been proposed that it was the material that formed a primitive cell membrane with a degree of impermeability during early evolution [50]. Such cells might have been hardly more than blobs of amyloid gel coating the interior of the inorganic compartments or aggregating to form large volumes with interstices between them acting as the intracellular medium.

Another key property of amyloid is self-recognition. Amyloid only forms between chains of identical, or nearly identical, peptides or proteins. This property, plus their stability, led Maury [61] to suggest they are inherently self-propagating and suited to being the earliest form of protein employed in organisms. The self-recognition implies that the side chains recognize one another. This presumably results from the hydrophobic moieties recognizing themselves as would the aromatic and uncharged hydrophilic entities. At acid pH values, aspartate and glutamate side chains could attract each other. Histidines might also attract each other when pH values are not too low. This leaves positively charged lysines and arginines, but at least they have fairly long side chains which might attract each other a little so that overall they would not be too repulsive. It is only easy to imagine this sort of self-recognition if the peptide chains are side by side and in the same orientation, i.e., in parallel rather than antiparallel β- or α-sheets [58,62].

### 2.6. Covalent Metal-Peptide Complexes

Copper, nickel, cobalt and iron cations readily complex with peptides in alkaline solutions by binding to successive main chain amide nitrogen atoms [63]. Amide NH protons are displaced by the metal during the formation of these tight complexes in which the main chain atoms adopt a flat conformation as seen in (Figure 2d). The angles are detailed in Table 1. Such complexes, with up to four amide protons substituted by metals, also occur naturally within a number of native proteins (prion protein of CJD) and enzymes (acetyl CoA synthase), sometimes at active sites. The Ni tetraglycine complex of Fig 2d exhibits a remarkable similarity to present day macrocyclic tetrapyrrole cofactors—a peptide pigment [64,65]. Also the analogous Co-tetraglycine resembles a corrinoid group [66]. In these types of complex the metal has octahedral coordination to four planar nitrogens with two positions free to assist in catalytic reactions. The peptide ones could thus have performed much the same catalytic functions as hemes and corrinoids, before being largely, but not entirely, superseded by them.

### 2.7. Cations Bind Niches and Catgrips

The niche [67] is a three to four residue motif with the characteristic ϕ, ψ angles given in Table 1. It is by far the commonest feature where main chain carbonyl groups bridge metals or partial positive groups. The niche accommodates atoms or groups that offer partial positive charges, including water molecules or metal ions, as well as amines, guanidines, and other NH_2_ groups. Seven percent of all residues in an average soluble protein belong to a niche; another 7% have the niche conformation but no obvious bridging group. Fifty-five percent of niches occur either following a type 1 β-turn or at the C-termini of α-helices, and niches are common C-terminal features of α-helices. 3/10 helices also frequently terminate in niches. Niches that bind K^+^, Na^+^ or Ca^2+^ occur in some functional contexts: in the cyclic peptides valinomycin and antamanide; in several enzymes that are allosterically activated by Na^+^ or K^+^; and in the calcium pump, where a niche is involved in the ion transport. Niches are of two sorts, the niche3 with three residues as in (Figure 2h) and the niche4 with four residues as in (Figure 2i). Unlike the other motifs described here these peptide motifs are not enantiomeric and cannot overlap.

Another enantiomeric conformation is called the catgrip [40] where alternating main chain CO groups bind Ca^++^ or other cations in a ring-shaped conformation as in (Figure 2g). The main chain CO groups point into the ring; this is employed for specific Ca^++^ ion binding in the annexin, phospholipase A2, and subtilisin loops, and the regularly arranged β-roll loops of the serralysin protease family. Apart from their role in calcium binding, catgrips are relatively common in proteins though their numbers are 10% or less compared to nests or niches.

### 2.8. The Earliest Peptides Using Side Chains? Metal-Carboxylate-Bound Nests

Many authors [6,68] have suggested that a limited number of easily-synthesized amino acids—perhaps glycine, alanine, aspartate and valine—were generated on the early Earth. A number of peptide stretches at functionally crucial and well-conserved positions in some key proteins including calmodulins, integrins and RNA and DNA polymerase, bind a metal ion directly via the three carboxylate side chains of a DxDGD sequence. Furthermore, all have an RL nest starting at the second aspartate residue. It is tempting to speculate that these various peptides are molecular fossils.

Calcium ions are important signaling molecules in most cells. The commonest proteins that bind calcium ions are the large family of canonical EF hand proteins [69] including calmodulin, which is found in eukaryotes, or its homologue calerythrin in prokaryotes. Typically they have the sequence DxDGD in which the three aspartate carboxylate groups bind a calcium ion (some have asparagines in place of aspartate). These carboxylate groups are, in turn, bound by the NH groups of an RLRL nest, beginning at the second aspartate [39]. The function of the nest is indirect in that it binds the carboxylates which bind the calcium ion. An advantage of such a sandwich arrangement seems to be that the calcium ion needs to continuously bind and diffuse away. When calcium is absent the carboxylates would be expected to repel each other and fly apart. However being bound to the LRLR nest discourages this.

Three of the blades of the extracellular β-propeller domain of the α subunit of α_IIb_β_3_ integrins [70] have calcium-binding DxDGD sequences (again, some with asparagines in place of aspartate). These carboxylates are bound by the NH groups of an RLRL nest, beginning at the second aspartate. The nests appear functionally similar to those in the EF-hands in binding the carboxylates which then bind the metal ions.

Both DNA-dependent and RNA-dependent RNA polymerases incorporate a DxDGD sequence at their active sites [71,72]. The sequence of the DNA-dependent ones are found in the β' subunit double-psi β-barrel; these enzymes are the ones employed in transcription to produce mRNA in all life forms: bacteria, archaea and eukaryotes. The RNA-dependent sequences [73] also occur in a double-ψβ-barrel; these enzymes are found in many, but not all, eukaryotes and some bacteriophages; their function is different, being concerned with the generation of double-stranded RNA to be processed via the RNA silencing system. In these proteins the aspartate side chains bind a magnesium ion which coordinates the phosphates of the substrate ribo- or deoxyribo- nucleotide at the catalytically active part of the active site. An RL nest occurs, beginning at the second aspartate; although the carboxylate-NH binding seen in the protein crystal structure is weaker than in calmodulin, the nest does bind the carboxylates, helping to hold them in place, particularly in the absence of Mg^++^.

The phosphoglucomutases, including phosphomannomutases, also have a DxDGD sequence binding either Mg^++^, Zn^++^ or Ni^++^ at the active site [74]. There is an RLR nest starting at the second aspartate. Presumably the function of this nest is similar to that of the other types of motif. Table 2 provides a sequence alignment of all these different motifs, including the positioning of the RL nest common to all these motifs.

**Table 2:** Homologous metal-binding sites in various proteins. All have an RL nest at the same position, shown in the top row. It is suggested that this structural feature is functionally useful in binding the carboxylate groups. RdRP means RNA-dependent RNA polymerase. DdRP means DNA-dependent RNA polymerase. PGM means phosphoglucomutase. PMM means phosphomannomutase.

**Protein**	**PDB no.**	**Subunit**	**Metal binding sequence**	**Metal**
Cons. nest			RL	
RdRP	1hqm	D	738 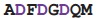 745	Mg
RdRP	1ibh	A	480 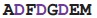 487	Mg
DdRP	2j7n	A	1006 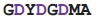 1013	Ca
calmodulin	1cdm		19 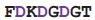 26	Ca
calmodulin	1cdm		55 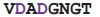 62	Ca
calmodulin	1cdm		128 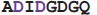 135	Ca
PMM	1p5d		241 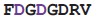 248	Zn
PGM	1kfi	A	307 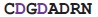 314	Zn
Integrin	3nid	C	296 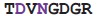 303	Ca
Integrin	3nid	C	364 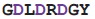 371	Ca
Integrin	3nid	C	425 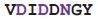 432	Ca
Cons. seq.			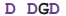	

### 2.9. Were Phosphate-Bound Nests Used in the Earliest Metabolisms?

**Figure 3:**
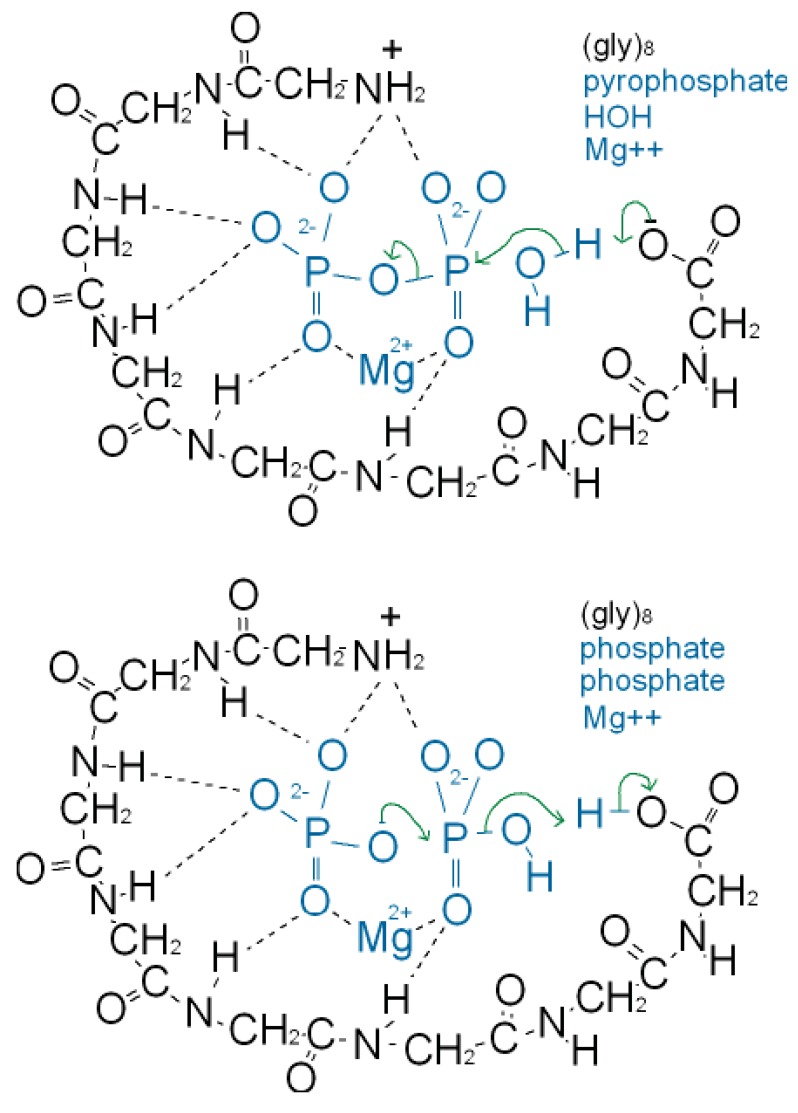
The envisaged pyrophosphate mechanism showing how the main chain atoms of short peptides may catalyze (**a**) the pyrophosphate reaction PPi → Pi + Pi and (**b**) the reverse reaction, pyrophosphatase synthase. The protonation states of the phosphates in (**b**) are uncertain. We have assumed that the proximity of the magnesium ion and the nest causes the reacting phosphate at the left to be PO_4_^3−^ while the reacting phosphate at the right is HPO_4_^2−^ or CH_3_COPO_4_^2−^ [75,20].

As well as binding phosphate, many P-loop proteins catalyze the transfer of a phosphate of ATP or GTP to HOH (ATPase, GTPase) or to a substrate (kinase). Some primitive anaerobic organisms use pyrophosphate, PPi, instead of ATP as the high energy molecule. It has been suggested that the earliest P-loop proteins made use of this energy by catalyzing the transfer of a PPi phosphate to HOH (PPase) as in (Figure 3a), and also enabled the reverse reaction of pyrophosphate synthase as in (Figure 3b) [75]. In the Figure it is supposed that the catalytic functions currently performed by side chain groups were originally carried out by main chain atoms. The C-terminal carboxylate acts as a base catalyst in the PPase reaction and an acid catalyst in the synthase reaction. In the event of an acetyl phosphate intermediate in the synthase reaction the right-hand phosphate in (Figure 3a) would be that of acetyl phosphate.

A present day protein that catalyzes the pyrophosphatase reaction is the integral membrane protein H^+^-pyrophosphatase or Na^+^-pyrophosphatase [76-78]. These proteins harness a protein or sodium gradient for pyrophosphate synthesis. This protein incorporates the sequence signature GxxxxGK characteristic of a P-loop [79] so probably has a phosphate-binding nest resembling that of other P-loop proteins. Accordingly the phosphate-binding part of this pyrophosphatase might qualify as being one of the most ancient relics of all. We argue that H^+^-pyrophosphatase was first on the grounds that the proton potential acting across the outer walls of the compartmentalized hydrothermal hatchery amounted to around 5 pH units or ∼300 millivolts [80]. As there is no measurable difference in sodium concentrations between the Lost City hydrothermal spring waters and the present day ocean [81] the Na^+^-pyrophosphatase may have developed later in highly alkaline saline conditions.

**Figure 4:**
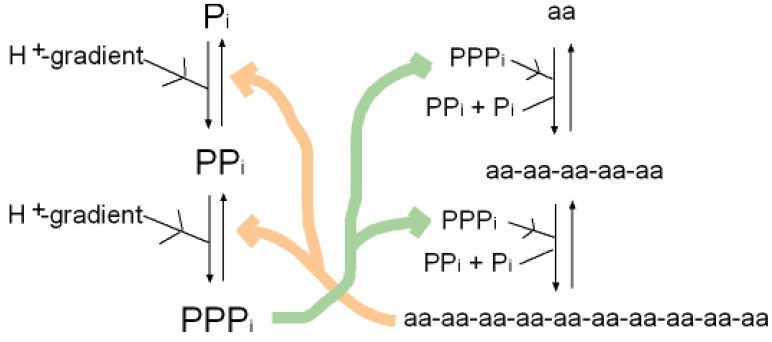
The synergy between short peptides and polyphosphates. Decapeptide aa-aa-aa-aa-aa-aa-aa-aa-aa-aa acts as an enzyme that, driven by a proton gradient (see Figure 1), phosphorylates phosphate to form pyrophosphate. The same enzyme phosphorylates pyrophosphate to form triphosphate. Triphosphate reacts with amino acids and phosphorylates them, giving rise to polypeptides after their dephosphorylation. In doing so triphosphate becomes hydrolyzed to phosphate and pyrophosphate. The effect is synergy between peptide and polyphosphates. Polyphosphates are required for making polypeptides which in turn catalyze polyphosphate formation. For the sake of simplicity, longer polyphosphates such as PPPPi and PPPPPi are not illustrated, but they may have played an active part in the process, just as longer peptides do [75].

A synergy that could form the basis of an early metabolism then emerges as shown in (Figure 4). Triphosphates have been shown to phosphorylate glycines and other amino acids chemically to form cyclic acylphosphoramidates that react with further amino acids to produce dipeptides. The dipeptides then react with such cyclic acylphosphoramidates to generate tripeptides and so on, forming higher oligopeptides [82,18-19,83]. In other words, polyphosphates react with amino acids to cause peptide formation. This, taken in conjunction with the previous suggestion, that P-loop peptides catalyze pyrophosphate, or polyphosphate, formation from phosphate, constitutes a self-perpetuating metabolic cycle that may have operated within the inorganic membranes comprising the exteriors of the sulfidic hydrothermal mounds (Figure 1).

## 3. Conclusions

Anion and cation binding tends to be regarded as mediated mainly by the charged side chains of aspartate, glutamate, lysine, arginine and histidine. This work shows that, in spite of the main chain atoms of peptides not being portrayed conventionally as having a formal charge, they are nonetheless often employed for binding anions and cations in current proteins. There are indications this was even more common in the earliest peptides when the side chains were presumably not genetically encoded and thus amino acids with charged side chains could not be relied upon to occur.

The anion-binding motif expected to have been particularly common in early evolution is the nest. This feature is present as a phosphate-binding feature within P-loops, which are well known as the most abundant ATP or GTP-binding motifs in proteins. In present day proteins P-loops are mostly associated with phosphoryl transfer reactions for energy generation from ATP or GTP and it seems plausible this function is an evolutionary relic of the earliest energy generating systems from di- or tri-phosphates. We also suggest a mutual symbiosis for the earliest form of metabolism emerging in a hydrothermal mound in which polyphosphates chemically react with amino acids resulting in peptide formation while the same peptides catalyze polyphosphate formation. Nests are also employed for binding iron-sulfur centers in proteins which would also have been catalytically useful in early evolution. In relation to nests the α-sheet conformation resembles a flattened version of nests. A key aspect for early evolution is that amyloid may have taken over from inorganic compartment walls as the first organic cell membranes. Some of the active inorganic ingredients were retained as protoenzymes—an advance on mere catalysis—allowing compartmentalized metabolism inside.

The commonest cation-binding motif is the niche and this was probably also present in the peptides during early evolution. Three other functionally useful cation-binding features are also described: (1) catgrips, for calcium binding; (2) peptidic channels, for sodium and potassium ion transport across membranes; and (3) covalent metal-peptide complexes where the peptide binds copper, nickel, cobalt or iron metal ions, for catalysis. A further cation-binding motif that seems likely to have evolved early on is the aspartate-containing DxDGD motif that is observed to bind specific metal ions at key functional binding sites of RNA polymerases, calmodulins, integrins and phosphoglucomutases. These employ the side chains of aspartate residues, which may have been synthesized relatively early on in evolution, in conjunction with nests.

Knowledge about the conformations of proteins and short polypeptides has changed. Some of the shapes of polypeptides likely to have occurred regularly in developing forms of life in an era leading to the RNA world can now be guessed. We begin to glimpse how the functional properties of these peptides, especially their facility for either cation or anion binding, would have assisted evolution of the earliest types of metabolisms. Of these, phosphate binding peptides appear to have left the most traces as they would seem to have been retained in a large proportion of proteins using ATP and GTP in present day proteins, occurring not only in a binding capacity but also still, arguably, catalyzing the energy-generating hydrolysis of these polyphosphates.
